# Reprogramming of the Genome-Wide DNA Methylation Landscape in Three-Dimensional Cancer Cell Cultures

**DOI:** 10.3390/cancers15071991

**Published:** 2023-03-27

**Authors:** Alma Jaqueline Heredia-Mendez, Gricelda Sánchez-Sánchez, César López-Camarillo

**Affiliations:** Posgrado en Ciencias Genómicas, Universidad Autónoma de la Ciudad de México, San Lorenzo 290, Colonia del Valle Sur, Ciudad de Mexico 03100, Mexico

**Keywords:** 3D cell cultures, epigenetics, DNA methylation, cancer

## Abstract

**Simple Summary:**

Genome-wide DNA methylation is a regulatory mechanism that is frequently altered in human cancers. Remarkably, these epigenetic marks on DNA are greatly influenced by microenvironmental factors, so to study them better, it is necessary to use novel cell cultures that faithfully reflect what happens in tumor cells in vivo. In this review, we highlight the importance of three-dimensional (3D) cell culture models, relative to traditional two-dimensional (2D) monolayer cell cultures, for the study of epigenetic DNA methylation in cancer. Moreover, we highlight how research approaches, by optimizing the 3D culture systems, can obtain a more realistic overview of the epigenetic landscape. The implications of the discovery of potential targets for cancer therapies are also discussed.

**Abstract:**

During the last century, 2D cell cultures have been the tool most widely used to study cancer biology, drug discovery, genomics, and the regulation of gene expression at genetic/epigenetic levels. However, this experimental approach has limitations in faithfully recreating the microenvironment and cellular processes occurring in tumors. For these reasons, 3D cell cultures have recently been implemented to optimize the conditions that better recreate the biological and molecular features of tumors, including cell–cell and cell–extracellular matrix (ECM) interactions, growth kinetics, metabolic activities, and the development of gradients in the cellular microenvironment affecting the availability of oxygen and nutrients. In this sense, tumor cells receive stimuli from the local environment, resulting in significant changes in their signaling pathways, gene expression, and transcriptional and epigenetic patterns. In this review, we discuss how different types of 3D cell culture models can be applied to characterize the epigenetic footprints of cancer cell lines, emphasizing that DNA methylation patterns play an essential role in the emergence and development of cancer. However, how 3D cancer cell cultures remodel the epigenetic programs is poorly understood, with very few studies in this emerging topic. Here, we have summarized the studies on the reprogramming of the epigenetic landscape of DNA methylation during tumorigenesis and discuss how it may be affected by microenvironmental factors, specifically in 3D cell cultures.

## 1. Introduction

Cancer cell cultures in 2D monolayers have been the tool most widely used in vitro to study the morphology and biology of cells, gene expression, molecular mechanisms in different environments or tumor stages, the mechanisms of action and response to drugs, and cancer stem cells’ biology [1].

Although the 2D cell culture model has served to elucidate several aspects of cancer’s biology, it has certain limitations, such as its growth in a monolayer attached to a plastic surface, which prevents cell–cell or extracellular matrix (ECM)–cell interactions from being recreated. These interactions are responsible for control of cell proliferation, viability, and differentiation. Another disadvantage is that in this type of culture, the cells have unlimited access to oxygen or nutrients from the medium, which does not allow the creation of concentration gradients, as observed in tumors in vivo [1,2].

In addition, 2D adherent cultures generally allow one to study only one type of cell, which results in the absence of a microenvironment or tumor niche where the cells grow; therefore, the cells’ morphology, polarity, and cell division are altered, which, in turn, modifies the organization of the cellular structures, cell signaling, the response to stimuli and drugs, gene expression, and epigenetic regulation. Therefore, a variety of 3D cell culture systems have been implemented that can be optimized to better mimic tumors’ physiology [2].

**Figure 1:**
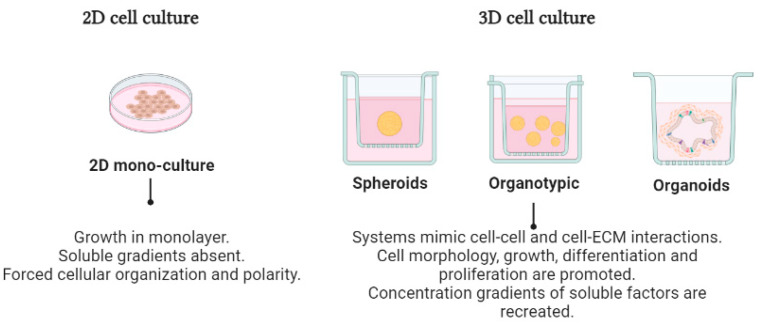
Characteristics of 2D and 3D cell cultures. Cells cultured in conventional 2D monolayers (**left**) exhibit a flat shape that does not accurately represent the morphology of the cells found in tumors. In contrast, cancer cells cultured in a 3D system (**right**) grow over a scaffold of ECM proteins that partially resemble the microenvironment, mimicking some cellular features of the in vivo condition; therefore, they are more appropriate for drug testing and gene expression studies.

Three-dimensional cell cultures were first developed in the 1970s by Hamburg and Salmon and reported in a seminal study which referred to the growth of cells in layers that formed spheroids or 3D structured masses that showed physiological functions, and physical and biochemical characteristics similar to those of tumors. (Figure 1) [1,3].

In 3D cell cultures, cell-to-cell and cell-to-ECM interactions are promoted; there is also the formation of nutrient, oxygen, and pH concentration gradients; and the growth kinetics are different, being greater at the periphery of the spheroids than at the bottom. In the center, a necrotic nucleus is formed [3]. In addition, the signaling pathways, responses to stimuli, patterns of gene expression, epigenetics, and metabolism are more similar to those of tumor cells. All these characteristics provide greater precision and sensitivity to the study of cancer cells and how the microenvironment influences their growth [3,4].

In this review, we focused on the impact of 3D culture systems on the epigenetic landscape of cancer cells. DNA methylation is one of the most important epigenetic marks that introduces significant changes in the gene expression of normal cells; however, its deregulation can influence the initiation and progression of human cancers. Several studies have shown that DNA methylation affects gene expression when normal patterns are altered, leading to the silencing of tumor suppressor genes or the re-expression of oncogenes [4]. However, it has been shown that this may change depending on the microenvironment and cellular context, which suggests that the effect of DNA methylation on gene expression is much more complicated than a simple on/off signal [5,6].

Tumor cells in the diverse stages of tumor development encounter different microenvironments that influence their cell fate and aggressive behavior. Due to their characteristics, 3D culture systems are a tool with great potential for studying the genetic plasticity that is known to modulate in vivo cellular phenotypes, but the underlying molecular mechanisms are poorly understood. For instance, the expression of the tumor suppressor E-cadherin gene can be controlled by microenvironmental and epigenetic factors during the epithelial–mesenchymal transition in the stages of tumor invasion and distant metastasis [7]. Breast cancer and 3D cultured squamous cell carcinoma tumor cells have been shown to experience a loss of E-cadherin expression due to DNA hypermethylation in its gene promoter region, which has been correlated with the transition from in situ to invasive cancer, metastasis, and poor prognosis in patients [7]. In other studies, it has been reported that the induced demethylation of E-cadherin led to its re-expression. Therefore, it is possible that the suppression of E-cadherin gene expression mediated by DNA methylation could be reversed and is related to heterogeneous expression patterns observed during the progression of cancer [7].

## 2. The Epigenetic Landscape Represents a Platform through Which Multiple Environmental Factors Interact

**Figure 2:**
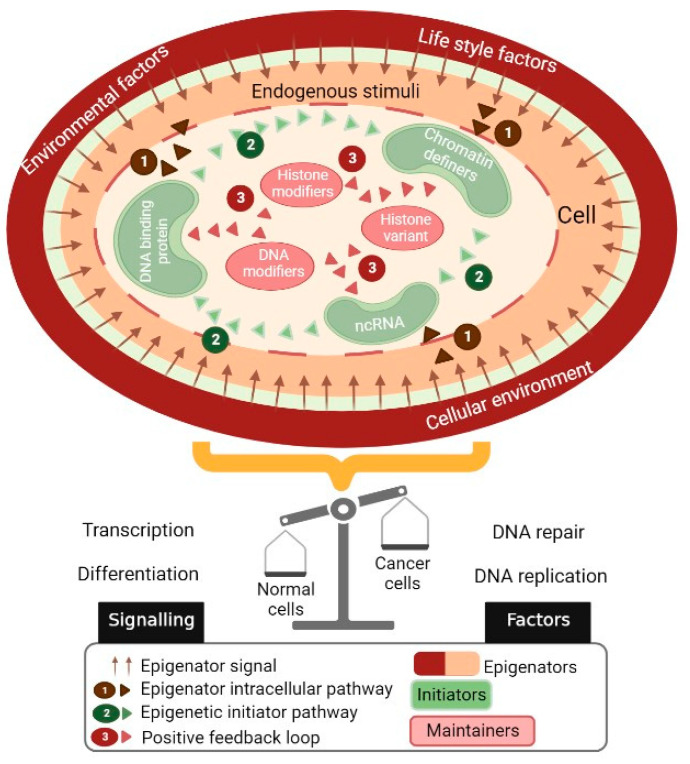
Influence of environmental factors on the establishment and maintenance of epigenetic states. The epigenators originate from the external environment of the cell, which activates specific signaling pathways. Epigenetic initiators respond to external signals that define the location of the epigenetic change within the chromatin. On the other hand, epigenetic maintainers support the epigenetic state of the chromatin. These modulators can dictate cellular outcomes by regulating key molecular processes including gene transcription, cell proliferation, and DNA repair, among others. Dysregulation of the epigenetic mechanisms by environmental and endogenous stressors may promote the development of abnormal phenotypes and cancer.

Epigenetic regulation, genetic modifications, and the organization of chromatin result in changes in gene expression without alterations in the DNA nucleotide sequence and can be inherited during cell division. Chromatin modifications and DNA methylation associated with noncoding RNA are the main epigenetic mechanisms that act to mediate reprogramming during the development and maintenance of cellular identity during the lifetime [8]. It is widely accepted that epigenetic modifications are stable; however, they can be further modulated by physiological, pathological, and environmental conditions (Figure 2). Previously, it has been reported that the epigenetic mechanisms can be altered in response to intrinsic, extrinsic, and environmental stimuli, resulting in the alteration of processes such as transcription, DNA repair, or the cell cycle, thus affecting the cell’s functions and contributing to the initiation and progression of cancer [8].

### 2.1. Remodeling of Chromatin by Histone Modifications

In the nucleus, DNA is condensed into a structure named chromatin, which is made up of units of nucleosomes. The structure of the nucleosome is made up of 147 base pairs of DNA that surround an octamer of globular proteins, namely, the histones, of which there are two oligomers of each subtype: H2A, H2B, H3, and H4. There is an external histone to the octamer, H1, which participates in the compaction of nucleosomes [9].

On each chromosome, chromatin is organized into different domains, such as euchromatin and heterochromatin, which are defined by their degree of compaction and association with gene functionality. Euchromatin is loosely compacted and allows gene transcription, while heterochromatin is more condensed and is associated with the repression of transcription. The main mechanism by which the structure and function of chromatin is regulated is through post-translational histone modifications that will later result in DNA methylation. There is a great variety of these modifications, especially in the N-terminal end of each histone that protrudes from the octamer. Among these, we can highlight the methylation and acetylation of lysine (K) and arginine (R), phosphorylation, glycosylation, ubiquitylation, and others. Enzymes that modify histones show different levels of specificity between different histones, as well as regarding the amino acid to be modified within the particular histone [9].

The histone code establishes a relationship between the patterns of histone’s post-translational modifications and the gene’s functionality, defining the state of transcriptionally active and inactive chromatin. Thus, histone modifications can directly alter the structure of chromatin and allow the DNA to access proteins that regulate DNA replication, repair, recombination, and transcription; they may also allow the binding of histone-modifying enzymes. Some of the histone modifications can be copied and propagated through the different cell divisions, contributing to the epigenetic inheritance of the transcriptional state [9].

### 2.2. DNA Methylation in Normal Cell Biology

DNA methylation is involved in phenotypic inheritance in single-celled organisms, as well as in transgenerational inheritance in multicellular organisms. The mechanism consists in the addition of a methyl group (-CH3) on carbon five of the pyrimidine ring of cytosine that is mediated by DNA methyltransferases (DNMTs). It occurs almost exclusively in cytosines that are located in dinucleotides with position 5′ CpG 3′, although there is evidence that it can occur in non-CpG dinucleotides such as CpA or CpT. In the genome, the CpG dinucleotides are distributed asymmetrically either in poor or dense regions called CpG islands; interestingly, 70% of the annotated genes are associated in their promoter region to CpG islands that are usually demethylated, whereas sporadic CpG sites in the rest of the genome are usually methylated [10,11,12].

**Figure 3:**
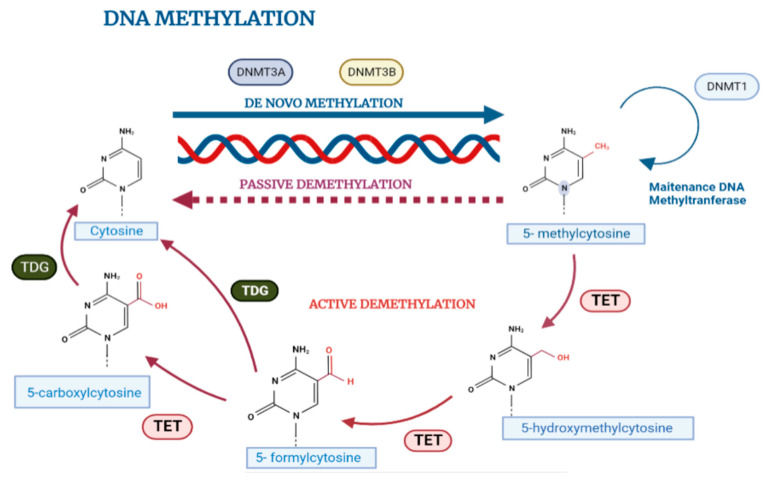
Maintenance and erasure of DNA methylation marks in normal cells. DNA methylation patterns are transmitted during DNA replication, where DNMT1 maintains global DNA methylation, whereas de novo methylation is performed by DNMT3A and DNMT3B proteins in complex with DNMT3L (not shown), a closely related homolog lacking a catalytic domain. DNA methylation marks can be deleted through a molecular mechanism in the absence of functional DNA methylation maintenance machinery during successive rounds of replication. On the contrary, the active demethylation of DNA occurs through an enzymatic process that eliminates or modifies the methyl group of 5 mC by the action of enzymes referred to as ten-eleven translocation (TET) enzymes.

In mammals, methylation patterns are established by DNA methyltransferase enzymes DNMT1, DNMT3a, and DNMT3b, which catalyze the transfer of the methyl group (-CH3) from an S-adenosil-L-methionine (SAM) donor group to cytosines in DNA (Figure 3). These enzymes can be divided into those that maintain or copy methylation marks after each round of cell division in DNA replication, and those that initiate new (de novo) methylation marks in DNA. The DNMT1 enzyme is the most abundant in somatic cells and is responsible for maintaining the methylation patterns required for correct embryonic development, inactivation of the X chromosome, and genomic imprinting; on the other hand, the methyltransferases DNMT3a and DNMT3b, in complex with DNMT3L, mediate de novo methylation and actively participate in establishing methylation patterns after embryo implantation. However, despite their specific functions, it has been shown that the three DNMT enzymes cooperate and participate in both maintenance processes and de novo methylation [11,12].

Cytosine methylation can be actively reversed to maintain balance in the human genome methylation profile by the family of methylcytosine translocation dioxygenase (TET) enzymes in a process whereby the sequential oxidation of 5-methylcytosine and TET 1/2/3 generates several intermediate groups (5-hydroxymethylcytosine, 5-carboxycytosine, or 5-formylmethylcytosine) which can then be demethylated via cleavage by DNA thymine glycosylase (TDG) and base excision repair (BER) mechanisms [13].

### 2.3. Influence of Microenvironment on DNA Methylation

Epigenetic variation over time may depend on genotype (intrinsic factors), environment (extrinsic factors), and stochastic factors (factors still indeterminate). In this sense, it is important to understand that the epigenetic state is specific to the tissue or cell, and that the degree of exposure to a given factor can determine the ability to induce specific epigenetic changes [14].

Many researchers have reported that epigenetic patterns differ significantly when examined in fresh tissue compared to in vitro culture conditions, and this is justified by the influence of the media and methods used for cell culture. Thus, it has been found, for example, that de novo methylation could silence nonessential genes in cell cultures, while demethylation in most cases has been reported to potentially provide a mechanism that influences the karyotype change of different normal cell lines to upregulate genes that confer a growth advantage [14].

## 3. Aberrant DNA Methylation Patterns in Cancer Cells

DNA methylation is a key process that takes place throughout development in multicellular organisms and ensures the maintenance of normal gene expression patterns. However, during the malignant transformation of normal into cancerous cells, a reprogramming of the epigenetic regulation is activated, which, combined with both genetic and environmental changes, contributes to the onset and progression of tumors [14].

**Figure 4:**
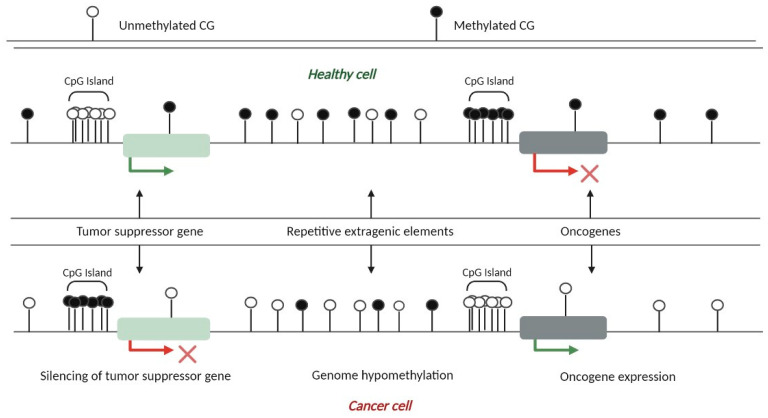
DNA methylation in normal and cancer cells. CpG dinucleotides present at a low density within the genomic sequence are mostly methylated (e.g., the silencing of transposable elements and the condensation of centromeric and subtelomeric regions). On the other hand, a small proportion of CpG dinucleotides in clusters named CpG islands are often found within the gene promoter regions. These CpG islands are rarely methylated in healthy cells. Aberrations in the epigenetic information in cancer cells are characterized by global genomic hypomethylation accompanied by aberrant hypermethylation of the CpG islands present in the promoter regions of tumor suppressor genes. The red arrow with the cross indicates the repression of transcription; the green arrow indicates active transcription. The green rectangle represents a promoter site for tumor suppressors and the gray rectangle shows a promoter site for oncogenes.

Aberrant patterns of DNA methylation are common in many types of human malignancies, including colon, stomach, cervix, prostate, and breast cancer, among others (Figure 4). In general, global hypomethylation in repetitive regions or transponible sequences, hypermethylation of gene-promoter regions, and mutagenesis of 5-methyl-cytosine sequences by spontaneous deamination of methylated cytosine are the most common alterations found in cancer. Thus, together, this series of alterations may contribute to the progression of the tumor [14].

Several studies have indicated that de novo DNA hypermethylation mediated by the DNMT3a and DNMT3B proteins in cancer cells specifically targets tumor suppressor genes, and that the degree of methylation varies among tissues, resulting in uncontrolled cell growth and proliferation [9,14,15]. Many tumor suppressor genes are inactivated by thismechanism, including adenomatous coli polyposis (APC), retinoblastoma (Rb), von Hippel–Lidau (VHL), BRCA1, and several other genes, such as those involved in DNA repair processes (MGMT, O-6-methylgunaine-DNA methyltransferase, hMLH1), cell adhesion (CDH1, TIMP-3, E-cadherin), hormone response genes (ER and PR), cell cycle progression (p16 INK4a, p15 INK4b), apoptosis (DAPK: protein kinase-1 associated with death), and antioxidation (GSPT1: glutathione-S-transferase P-1) [9,11].

Global hypomethylation also contributes to genomic instability and the rupture of DNA, which is often accompanied by loss of the imprint of some oncogenes, such as urokinase protease, mesothelin, claudin, heparinase, E-cadherin, proopiomelanocortin (POMC), and S100A4 (S100 calcium-binding protein A4), which leads to the development of cancer. Global hypomethylation has been attributed to three main causes: (1) a lack of coordination between DNMT-1′s activity and DNA replication, (2) a selection of hypomethylated DNA patterns accompanied by the overexpression of specific oncogenes or genomic instability that facilitate the growth and expansion of cancer cells, and (3) a consequence of alterations in the organization of chromatin and the cell disorganization produced during the progression of cancer [14,15].

Under normal conditions, epigenetic profiles in the cells maintain a balance between the processes of DNA methylation and demethylation. However, this balance is altered under pathological conditions such as inflammation, oxidative stress, and cancer, resulting in diverse aberrant phenotypes. It has been reported that DNA methylation can adapt to these environmental factors in cancer, resulting in modifications in the global methylation patterns or specific CpG sites, and by inducing the formation of methyl group donors or by modifying the activity of DNMT or TET enzymes [15,16].

## 4. Three-Dimensional Cell Cultures in Cancer Research

An increasing amount of new evidence has shown that, compared with 2D cultures, 3D cell cultures are physiologically more relevant to studies of morphology, cell number monitoring, proliferation, response to stimuli, differentiation, and drug efficacy (Table 1). This is because 3D culture conditions can be optimized to better recreate the tumor microenvironment characterized by cell–cell and cell–extracellular matrix interactions. In addition, cells cultured in 3D better reproduce the development of gradients of O_2_ and nutrients, as well the activation of cell signaling pathways and the specific gene expression patterns driven by the ECM. Moreover, the epigenetic patterns responsible for the polarity and morphology of tumor cells in vivo are also mimicked in 3D cell cultures [16,17]. Most 3D cell culture systems are based on the utilization of natural or artificial solid scaffolds (organotypic), or scaffold-free systems (spheroids) which are grown in a suspension over plastic substrates. However, it is of utmost importance to consider, before choosing the study system, which would be most relevant and appropriate for the purposes of the research to be performed.

### 4.1. Organotypic 3D Cell Cultures

In 3D organotypic cultures, cancer cells are grown to make 3D spheroid-like structures over semisolid supports that can be based on hydrogels or natural hard polymeric materials of animal or plant origin. Collagen-based hydrogels with proteins such as laminin, proteoglycans, and glycoproteins are the most often used because of their mechanical properties, which can be reconstructed in a structure like that of the native extracellular matrix; added to this, the presence of growth factors, such as vascular endothelial growth factor (VEGF), platelet-derived growth factor (PDGF), and hepatocyte growth factor (HGF), which can be integrated into this type of culture, makes this type of methodology very suitable for easily recapitulating many aspects of tumors’ physiology as well as the nutrition and oxygen gradients seen in tumors in vivo [23].

### 4.2. Scaffold-Free 3D Systems (Spheroids)

The self-assembly of cells and tissues is a natural phenomenon during organogenesis and morphogenesis. However, in 3D cell cultures, the scaffolding that allows individual cells to self-assemble and form large aggregates, known as spheroids, is not used. By general definition, spheroids are cellular aggregates that grow in 3D in a suspension; they can be homotypic, formed only by cancer cells, or heterotypic cells formed by cancer cells and other cell types. The cells form spherical structures with a balanced morphology of variable size (50, 150, or up to 500 μm), which is formed by a necrotic nucleus or resting cells and a peripheral layer with proliferating cells [6,16,23]. Historically, in 1944, Holt Freter was the first to use spheroids as a morphogenetic model in his research into skin’s behavior during development; for cancer research, this multicellular tumor model was initially created in the early 1970s and was applied for diverse types of cancers in vitro [6,24,25]. Currently, for the generation of spheroids, a great diversity of techniques can be used, including the use of microplates of hanging drops, magnetic levitation, spheroidal microplates with ultra-low fixing coating, and cultures in bioreactors [23].

### 4.3. Organoid-Type Culture Systems

Organoids are 3D cultures developed from stem cells, which create a favorable artificial environment in which cells can grow and interact in a three-dimensional environment like the conditions of an in vivo state, mimicking their molecular functioning and preserving the tissue’s original structure [24,25].

Organoids are produced from one or a few pluripotent stem cells (PSC), including embryonic stem cells (ESC), induced pluripotent stem cells (iPSC), tissue stem cells, adult stem cells (ASC), or tumor stem cells, which are characterized as being pluripotent; that is, in addition to growing in vitro and having the ability to “differentiate”, they can generate specialized cells of different types, similar to those that form real organs [16,24,25].

In general, and whatever the starting point, the cells are placed in a culture medium that contains the nutrients and growth factors that have an important function for the development of a specific organ. Gel is then added with substrates like those of the extracellular matrix to allow a porous structure to form, which serves as a support for the cells’ growth. In this way, the cells eventually form spherelike structures that float in the middle and can be maintained for an indefinite time. These spheroids can be differentiated spontaneously or by induction, according to the lineages or the desired cell type, by adding or removing specific differentiating factors. Although organoid systems have been very useful, one of the major drawbacks of this type of culture is the lack of reproducibility due to tumor heterogeneity; for example, for organoids derived from iPSC, the disadvantages lie in the fact that their effectiveness depends on the type of cancer and the presence or absence of oncogenic mutations that confer advantages for the growth of the tumor’s subclones [16,25].

Although 3D cell cultures have shown clear advantages and seem to be more appropriate for studying cancer cells’ biology and responses to novel drugs in comparison with 2D monolayer cell cultures, they also display several disadvantages, which have been previously reported [1,24,25,26]. These are summarized in Table 2.

## 5. Three-Dimensional Cell Cultures and Microenvironmental Factors as Key Regulators of Epigenetic Programs in Cancer

For many years, the study of cancer has been carried out using cell cultures that have poorly approximated tumors’ heterogeneity. A fundamental assumption underlying the use of monolayer 2D cell cultures for biological research is that the cell lines can retain the molecular characteristics of the tissues from which they derive, allowing in vitro observations to be translated directly into an in vivo context. However, more than 30 years ago, different research groups, such as those headed by Antequera, Boyes, and Bird, reported differences in the DNA methylation patterns in 14 loci containing CpG islands in the mouse NH3T3 and L cell lines, interpreted as cell culture-induced changes in DNA methylation occurring in non-essential genes [16,26]. After these early findings, they performed a genomic scanning assay by reference restriction to compare six human embryonic stem cell lines cultured under different conditions. Overall, their results indicated that there were significant differences in the DNA methylation patterns induced by cell cultures. In this case, these changes were attributed to the serum content in the culture medium used, thus reporting one of the first pieces of evidence that the microenvironment can influence changes in the tumor’s epigenetic landscape.

Although the functional roles of microenvironmental factors that may influence the modification of the epigenetic landscape during the progression of cancer are still unclear, there are several reports about the urgency of creating novel cell culture models that are more representative of tumors’ physiology in vivo. Therefore, with the application of 3D culture methods, some studies have been carried out to investigate whether these systems exhibit the ability to better reflect tumors’ epigenetic plasticity. DesRochers and coworkers documented that in 3D cultures of skin cancer cells, the expression of E-cadherin was dynamic and sensitive to the induction of complex homotypic cell–cell interactions induced by changes in the DNA methylation patterns [8].

Additional evidence of the key role of 3D cell cultures in epigenetic regulation comes from Amatangelo and collaborators, who found that the use of a specific histone methyltransferase inhibitor, EZH2, resulted in inhibited cell growth, invasion, and the induction of apoptosis in epithelial ovarian cancer cells cultured under 2D and 3D conditions. Remarkably, the effects of the epigenetic inhibitor in the cancer hallmarks were specifically observed in 3D cultures and could be associated with changes in the pattern of DNA methylation. This has important clinical implications, as 3D cell cultures, in contrast to 2D monolayers, were able to demonstrate the sensitization of epithelial ovarian cancer cells to inhibition by EZH2 methyltransferase [26].

On the other hand, Dumont and colleagues showed that when cells grown under 2D conditions were cultured in an environment that induced the epithelial-to-mesenchymal transition (EMT), they acquired de novo DNA methylation at specific sites in the genome. The repression of the expression of E-cadherin preceded the subsequent acquisition of methylated CpG sites. Moreover, the repression of p16 INK4A signaling in primary human mammary epithelial cells (HMEC) activated an E2F-mediated increase in chromatin-remodeling proteins and caused de novo DNA methylation in a non-random collection of loci in cells cultured under 3D conditions with exposure to different serum levels. These findings showed that cells can acquire genetic plasticity by alternating the p16/pRb pathway and that this de novo methylation program has a predictable rather than a random pattern [27].

Nestor and coworkers conducted another interesting study, focused on evaluating the effects of adding enzymatic cofactors that are necessary to increase the activity of TET enzymes to the culture media. Vitamin C and 2-oxoglutarate act as cofactors to efficiently catalyze the hydroxylation of 5-methylcytosine (5mC) in canonical CpG dinucleotides to 5-hydroxymethylcytosine (5hmC). The data indicated that by supplementing the culture media with these cofactors in cell lines from different types of cancer in which TET enzymes had low levels or no activity, a partial but substantial recovery of the overall levels of 5hmC was possible because of the increasing the activity of TET enzymes. Together, these results suggested that the observed loss of 5hmC resulted both from a reduction in TET enzyme levels and from an overall loss of TET enzyme activity due to the limiting cofactors [19,28].

## 6. Three-Dimensional Cell Culture Models Allow a Better Study of the Epigenetic Landscape in Cancer

During tumorigenesis, the generalized changes in DNA methylation can be quite variable within tumor cells, and this may be due to the influence of the tumor microenvironment, suggesting that epigenetic plasticity may be related to the phenotypic heterogeneity of cell populations that are present in both primary and metastatic tumors. Therefore, it is extremely important to make use of optimized in vitro models with the ability to reproduce the tumor microenvironment in vivo [29].

It is important to emphasize that the influence of the cellular microenvironment plays an important role in epigenetic plasticity. DNA methylation is influenced by factors such as cell–cell and cell–extracellular matrix interactions in the context of tissues, and this leads to alterations in the methylation profiles silencing or overexpressing genes such as E-cadherin, which play a very important role in the processes of disseminating and acquiring a migratory phenotype that is dependent on the epithelial–mesenchymal transition (EMT) of tumor cells, the existing evidence showing relationships among the variations. The expression of this gene has been associated with the microenvironment during the different steps of metastatic and invasive processes in the progression of cancer. Thus, it has been shown that E-cadherin is generally silenced by promoter hypermethylation in 2D cultures; however, in 3D cultures that reproduce cell–cell and cell–extracellular matrix interactions, E-cadherin is re-expressed by hypomethylation of the promoters. This makes sense of the need for cells to form metastases to re-establish the adhesions measured by E-cadherin to reverse the migratory phenotype and allow the formation of premetastatic niches [29,30].

Interestingly, the levels and activity of DNMTs have also been analyzed, and it has been suggested that proteins such as laminin-1 and various microRNAs inhibit their activity in 3D cultures. It is possible, then, that DNA methylation in tumors is altered due to processes related to microRNAs, which may also be regulated by hypo- and hypermethylation [31].

On the other hand, research has been carried out on cancer stem cells, which are cells with high tumorigenicity that have high potential for renewal and play a fundamental role in tumor initiation and metastasis. In this sense, it is believed that DNA methylation has an important role in regulating the expression of genes such as OCT4, NANOG, SOX2, and KLF4, which are essential for maintaining stem cells’ characteristics. Significant changes have been reported when this process has been studied in 2D- and 3D-cultured cells, the results obtained regarding altered methylation patterns in 3D cell cultures were more comparable with tissue-based analysis [32,33,34].

Epigenetic processes are very complex, and how the microenvironment participate in the regulation of these continues to be a very important issue to elucidate. This section addresses some 3D culture systems that have been used as biological tools in several investigations (Table 3) to better reflect the tumor epigenetic landscape in vitro.

### 6.1. Alterations in DNA Methylation in Organotypic 3D Cell Cultures

Cells can detect extracellular stiffness by binding the membrane’s beta-integrin proteins to the ECM, resulting in the subsequent activation of actomyosin’s contractility, rearrangements of the cytoskeleton around the cell membrane, and the activation of mechanosensitive proteins and signaling pathways such as lamina, talin, RhoA 1, Rac 1, focal adhesion kinase (FAK), and PI3K. Together, these events regulate the localization and activation of transcription factors. Therefore, many studies have highlighted the influence of the rigidity or softness of the ECM on changes in the epigenetic landscape. Thus, it has been proposed to optimize 3D culture systems to control the aforementioned factors, among others, and thus better reflect what happens in tumors in vivo [20,33,34].

Stowers and collaborators also proposed using reconstructed basal membrane matrices with Type I collagen and alginate as systems in which the rigidity of the extracellular matrix can be adjusted. It is important to note that more rigid matrices have resulted in cellular morphologies and malignant phenotypes in breast, liver, pancreas, lung, and brain cancers compared with less rigid matrices [35,36]. Thus, it has been reported that improvements in the rigidity of the extracellular matrix can exacerbate traits such as cell migration and invasion, reflecting significant changes in the epigenetic landscape, especially in DNA methylation.

### 6.2. Cancer Co-Culture Models for Studying DNA Methylation

There is a great need to define the microenvironmental factors that play a key role as regulators of tumor’s epigenetic plasticity for the development of optimal 3D systems that mimic the complexity observed in vivo.

Tumor cell co-cultures have been proposed for studies of the epigenetic landscape. For example, Kaur et al. proposed using matrices enriched with neutralized Type I collagen to which human dermal fibroblasts were added as a basis for skin cancer cell growth. By analyzing these cultures, they were able to identify that there were significant changes in the DNA methylation patterns and even in the expression of the chromatin remodeling proteins compared with 2D cultures [37]. Significantly, this research group corroborated the great similarity that existed in the DNA methylation patterns between fresh tissue samples and cells grown in the co-culture system.

### 6.3. Alterations in DNA Methylation Patterns in Organoid-Type Cell Cultures

Organoids can be defined as 3D structures derived from stem cells that mimic the cell types and self-organization present in in vivo samples. It has been proposed that human cancer organoids generally exhibit DNA methylation profiles that are closer to those of primary tumor samples than cancer cell lines established in 2D cultures. The exceptional advancement of organoids derived from epithelial cells, originally described in intestinal cells, was to achieve the inactivation of the WNT pathway and inhibition of the BMP pathway. This procedure is now applied to breast, lung, liver, pancreas, and other cancer cells, allowing the growth of normal tissues and the corresponding tumor types [38,39].

In 2020, Joshi and collaborators performed comparative analyses of the DNA methylation patterns of cells derived from primary colorectal cancer tumors and pancreatic, esophageal, and stomach cancer cultures of the organoid type. They demonstrated that the biological systems (organoids) maintained the characteristic epigenetic signature of the original primary cancer type [38,39]. The advantages of human cancer organoids are the efficiency of deriving the most common epithelial tumor types, the possibility of growing part of the normal tissue neighbor, the relative preservation of intratumor heterogeneity, and their versatility for genetic manipulation and drug testing [39,40].

## 7. Conclusions

During early development and cell differentiation, the entire genome is reprogrammed through epigenetic modifications, such as DNA methylation, histone modification, and interactions of non-coding RNA, which alter chromatin’s structure and DNA access through establishing a differential gene expression program specific to each cell without changes in the DNA sequence. Epigenetic reprogramming is essential for normal development as well as for the maintenance of cell-type-specific epigenetic patterns during cell division. However, given the dynamic and reversible characteristics of epigenetic modifications, epigenetic reprogramming is strongly affected by environmental factors that play a fundamental role in the establishment and maintenance of epigenetic markers [41].

Aberrant epigenetic reprogramming is associated with developmental disorders, such as imprinting defects and multifactorial diseases, which may include cardiovascular diseases or metabolic syndromes. Due to its ability to regulate cell growth and differentiation pathways, non-mutational epigenetic reprogramming has been added as a hallmark of cancer and perhaps as a driving mutational event in sporadic cancers favoring genomic instability, tumor initiation, and malignant progression. These epigenetic changes confer a specific phenotype to cancer cells, such as uncontrolled growth, resistance to cell death, and greater invasive and metastatic capacity.

The deregulation of or effect on the activity of the epigenetic machinery leads to the loss of global epigenetic marks, the activation of genes related to growth (oncogenes), and the silencing of genes that participate in the control of the cell cycle (tumor suppressors) and DNA repair [41]. These epigenetic features are like those seen in early development, where somatic cells are reprogrammed to a less differentiated state, followed by oncogenic reprogramming. The stem cell state in the development of cancer is one of the major challenges for treatment, as it promotes unlimited self-renewal, multilineage differentiation and drug resistance, alterations in the tumor microenvironment, tumor heterogeneity, and regulation of stromal cells associated with functional capacities acquired through epigenetic reprogramming.

Here, we have summarized studies on the reprogramming of the epigenetic landscape of DNA methylation during tumorigenesis and how it may be affected by microenvironmental factors, specifically by using 3D cell cultures as a model. Typically, most cancer studies have used 2D tumor cell cultures, which have a few limitations in reflecting what occurs in a tumor in vivo. Emerging evidence has suggested that aberrant epigenetic changes, such as DNA methylation, represent an important mechanism driving the initiation and progression of tumors. Due to its dynamic nature in response to physiological changes and microenvironmental stimuli, DNA methylation has served as a potential biomarker associated with cancer prognosis, drug resistance, and the progression of cancer [42,43,44,45].

The current studies on this topic have highlighted the importance of microenvironmental factors in epigenetic reprogramming during tumorigenesis and drug responses in cancer. We also illustrated how some researchers have optimized different 3D cell culture methods to more closely mimic the epigenetic changes that occur in a tumor in vivo and, thus, have adopted a more accurate approach during the study of different hallmarks of cancer. Moreover, this type of culture system will allow the recreation of more reliable platforms for more successful development of specific and targeted epigenetic therapies and treatments against cancer. These assumptions are based on recent advances which have shown the extensive reprogramming of the epigenetic landscape due to microenvironmental changes leading to aberrant gene expression patterns that contribute to the malignant progression of cancer. However, despite remarkable advances, further research is required to uncover which is the best cell culture model to reflect every single component of the tumor microenvironment involved in the epigenetic landscape [46,47].

In summary, the evidence highlights the importance of the microenvironment and a 3D architecture in the epigenetic modifications of tumor cells. Moreover, the experimental evidence has indicated that there is a growing need to optimize the conventional cell culture systems to accurately reflect the morphology of tissues and thus better understand the effects of 3D cell culture conditions on the dynamic cancer epigenome. The use of 3D cultures will permit us to better characterize the hallmarks of cancer and the therapeutic response to better and more rapidly translate basic results into clinical and personalized therapies in cancer.

## Figures and Tables

**Table 1:** Comparison of the different types of 3D and 2D cell culture models.

2D Cell Culture		3D Cell Culture			References
		Organotypic	Spheroids	Organoids	
**Cell** **morphology**	Flattened morphology Monolayer polarized	Including rounder, spread out, and elongated preserved natural cell structures	Rounded spheroids Contain multiple layers	Aggregate form Spherelike structure High polarity	[18]
**Cell–cell** **Interactions**	Cell–cell and cell–extracellular environment interactions are not represented	Cell–cell and cell–ECM interactions	Strong cell–cell connections Tight intercellular connections	Cell–cell and cell–3D matrix interactions	[19]
**Culture** **quality**	Highly reproducible Easy to interpret Simplicity of culture	Ability to mimic the ECM High sensitivity to drugs and reproducibility Variety of matrigels with high mechanical-structural differences	High reproducibility and sensitivity to drugs Moderate mechanical-structural integrity of the cells	Highly reproducible. High organotypic differentiation and in vivo-like functions	[20]
**Molecular mechanisms**	Loss of differentiated function, lacking tissue-specific environments and the ECM	Morphology and gene expression patterns, but also migration, cell cycling, and proliferation	Mimic the vascular structure of native tissues. Cells are in contact with the ECM Diffusion gradient of nutrients, waste, oxygen, and drugs	Interactions, mechanical cues, and features such as fluid flow, shear forces, stretching, and organ–organ interactions	[20,21]
**Drug** **response**	Low sensitivity to drugs	Depending on the 3D environment, the culture might become resistant or susceptible to drugs	Variable resistance to drugs	Depending on the 3D environment, the culture might become resistant or susceptible to drugs	[22]
**Applications**	Therapeutics for diseases	In vitro angiogenesis and drug testing, drug response studies for cancer research	Disease modeling and regenerative medicine Target identification and validation using RNAi	Therapeutic drug screening as well as personalized medicine	[23]

**Table 2:** Comparison between 2D versus 3D cell cultures: Advantages and disadvantages.

Type of System	Description	Advantages	Disadvantages
**2D cell cultures** 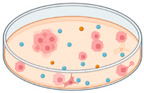	Cells grow on flat dishes, regularly made of plastic, where they adhere and spread until they reach confluence.	Inexpensive Ease of observing cells for interpretation Cells can be easily extracted from the medium and used for further experiments Most frequently used method in laboratories	Are not representative of real cellular environments Consist of individual dispersed cells Loss of the original tissue’s heterogeneity Lack of nutrients and oxygen gradients Homogeneous exposition to nutrients and to drugs Lack of ECM–cell interactions and signaling activated by the substrate
**3D cell cultures**			
**Spheroids** 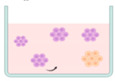	Spheroids grow over plastic surfaces forming floating 3D structures in which the cells form various layers which mimic some of the physical and biochemical features of solid tumor masses.	Can better to mimic a tumor mass Better at recreating the cell–cell interactions in different types of tumor cells Establishment of barriers between tissues Formation of nutrients and oxygen nutrients	More expensive and time- consuming Few commercially available Lack of nutrients at the core of spheroids affecting cell viability Lack of ECM–cell interactions and signaling activated by the substrate’s compounds
**Organotypic cell cultures in scaffolds** 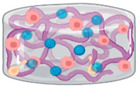	A 3D scaffold provides a way for cells to grow in three dimensions on a 3D plate. It has the ability to mimic the microenvironment in vivo more closely. These are typically provided through biomaterials of animal or vegetable origin called “hydrogels” as an ECM in which cells can survive, grow, and proliferate.	Can be accurately grown and measured An ideal environment for drug discovery and development The high levels of viability in cells can promote cell–cell and cell–ECM interactions and further affect the cells’ shape, metabolism, function, migration, proliferation, differentiation, and adhesion	Natural ECM has poor mechanical properties High sensitivity to enzymes, which limits its potential for application Scaffolds and the topography of cell distribution may cause various behaviors of the cell Microscopic cell observations and cell extraction are restricted for some analyses
**Organoids** 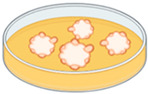	The reason for growth in 3D organoid cell cultures is that it is simply a better way of representing human tissues outside the body.	Organoids are a more realistic way to grow and treat cells, imitating the architecture of the parental tissues They are suitable for gene editing and used for simulating host–microbe interactions They can self-renew and maintain the physiological structure and function of tissues Creation of organoid biobanks becomes possible and, in this way, reduces the use of animal models	More time-consuming and very expensive Lack of high-fidelity cell types The limited maturation, atypical physiology, and lack of realization are features that may limit their reliability for certain applications

**Table 3:** A summary of studies examining epigenetic regulation in 3D cancer cell cultures.

Reported Studies	References
Rapid reprogramming of epigenetic and transcriptional profiles in mammalian culture systems	[19]
Methylation changes of primary tumors, monolayer, and spheroid tissue culture environments in malignant melanoma and breast carcinoma	[21]
Spheroid culture system methods and applications for mesenchymal stem cells	[23]
Three-dimensional culture sensitizes epithelial ovarian cancer cells to EZH2 methyltransferase inhibition	[26]
Dynamically stiffened matrix promotes malignant transformation of mammary epithelial cells via collective mechanical signaling	[32]
Matrix stiffness induces a tumorigenic phenotype in mammary epithelium through changes in chromatin accessibility	[34]
Dynamically softened substrate regulates malignancy of breast tumor cells	[35]
Remodeling of the collagen matrix in aging skin promotes melanoma metastasis and affects immune cell motility	[36]
Current concepts in tumor-derived organoids	[37]
The DNA methylation landscape of human cancer organoids available at the American type culture collection	[38]
Scaffold-free 3D cell sheet technique bridges the gap between 2D cell culture and animal models	[39]
